# Goal Achievement in 3017 Patients at Very High Cardiovascular Risk Based on Different LDL Cholesterol Calculations and Non-HDL Cholesterol Levels—Shortcomings of the Use of Non-HDL Cholesterol as a Target Depending on Triglyceride Levels

**DOI:** 10.3390/jcm14145003

**Published:** 2025-07-15

**Authors:** István Reiber, Laszlo Mark, Hajnalka Lőrincz, Ferenc Együd, Izabella Mező, György Paragh

**Affiliations:** 1 Department of Medicine, St George University Teaching Hospital of Fejer County, 8000 Szekesfehervar, Hungary; 2 Department of Cardiology, Bekes County Central Hospital Pandy Kalman Branch, 5700 Gyula, Hungary; 3 Division of Metabolism, Department of Internal Medicine, Faculty of Medicine, University of Debrecen, 4032 Debrecen, Hungary; 4 Gedeon Richter Plc., 1103 Budapest, Hungary

**Keywords:** rosuvastatin, statin–ezetimibe combination, LDL cholesterol, non-HDL cholesterol, target values

## Abstract

**Objectives**: The goal of this study was to investigate lipid goal achievement rates in very high-risk patients over six months using high-intensity rosuvastatin or rosuvastatin/ezetimibe combination lipid-lowering therapy. **Methods**: This prospective, observational study was conducted on the patients of 150 general and 60 specialist practices. Our analysis included 3017 patients (47% women) who completed six months of therapy. Of these, 55.5% had pre-existing cardiovascular disease, 35.6% had cerebrovascular disease, and 20.4% had peripheral vascular disease. **Results**: At six months, in patients receiving rosuvastatin monotherapy (20.5%), the 1.8 LDL-C achievement rate was 37%; in those taking the fix rosuvastatin/ezetimibe combination (63.7%), it was 52%, and the 1.4 level attainment proved to be 11% and 22%, respectively. The rates of LDL-C reduction of at least 50% were 32% and 42%, respectively. Overall, non-HDL-C goal achievement rates were higher than when LDL-C was calculated using the Martin–Hopkins or Sampson methods but similar to those calculated with the Friedewald formula. When patients were stratified by triglyceride quartiles, non-HDL-C goal achievement rates were significantly higher (*p* < 0.001) in cases with triglyceride levels below 1.2 mmol/L. Conversely, Friedewald-calculated LDL-C (F-LDL-C) goal achievement rates were significantly higher (*p* < 0.001) in patients with triglyceride levels above 1.7 mmol/L. **Conclusions**: Our findings suggest that the consistent use of fixed high-intensity statin and ezetimibe combinations can improve lipid goal achievement. However, comparing the achievement of LDL-C goals (calculated by three methods) and non-HDL-C goals also confirmed that the common practice of automatically adding 0.8 mmol/L to the calculated LDL-C value to determine non-HDL-C leads to inaccuracies, particularly in the lower triglyceride ranges.

## 1. Introduction

Cholesterol is not only a risk factor but also a primary driver of atherosclerotic vascular lesions through its deposition in vessel walls [1]. The understanding of this began to solidify in 1984, with the publication of the Lipid Research Clinics Coronary Primary Prevention Trial (LRC-CPPT) with cholestyramine, demonstrating that lowering cholesterol levels could significantly reduce the incidence of cardiovascular events [2]. A decade later, in 1994, the landmark study of modern lipidology, the Scandinavian Simvastatin Survival Study (4S), was published, which showed that simvastatin was able to significantly reduce cardiovascular events and all-cause mortality in a cohort of 4444 patients [3]. Over the past three decades, extensive clinical trials have established lipid-lowering therapy as one of the most successful and evidence-based strategies in cardiovascular prevention.

Globally, the effectiveness of lipid-lowering therapy is routinely monitored by measuring LDL cholesterol (LDL-C) levels. It is well known that apolipoprotein B 100 (ApoB) would be a superior marker for assessing vascular risk, although its measurement is not yet routinely available in everyday clinical practice [4]. More easily calculable non-HDL cholesterol (non-HDL-C) would be useful for determining apoB-containing lipids, particularly in cases with elevated triglyceride levels, such as diabetes, and its uptake in practice could be greatly aided by the inclusion of non-HDL-C as an important factor in the SCORE2 risk classification system within the European Society of Cardiology’s 2021 guidelines for cardiovascular prevention, which is likely to significantly promote its adoption in clinical practice [5].

The effectiveness of lipid-lowering treatment is typically monitored by the achievement of LDL-C goals, for which the most ideal measure would be direct LDL (D-LDL). The gold standard for D-LDL-C determination is the ultracentrifuge method, but this is not practical for routine clinical use. Instead, various homogeneous enzyme methods are employed. Unfortunately, for financial reasons, laboratory D-LDL-C is not accessible to all healthcare professionals treating dyslipidemia patients in many regions, including Hungary.

In the absence of D-LDL-C, we are forced to calculate LDL-C, the traditional method of which is the Friedewald formula, which has been in use since the 1970s and has been known for some time to be severely limited in its usefulness, especially for triglyceride (TG) levels above 2.3 mmol/L and in the LDL-C range below 1.8 mmol/L [6]. In 2013, SS Martin et al. published a new LDL-C calculation method (Martin/Hopkins calculation, MH-LDL-C), which is virtually a modified Friedewald formula [7]. The key difference is that instead of a universal number used in the Friedewald formula (2.2 for mmol/L), TG is divided by a modified number determined by non-HDL-C and TG values, i.e., the LDL-C bias of TG is less pronounced.

More recently, another non-direct method for determining LDL-C, developed by Sampson et al. (S-LDL), has emerged. This method correlates well with the determination of LDL-C by beta quantification and can be used for TG levels ranging from above 4.5 mmol/L up to 9.03 mmol/L [8]. An extended version of the Martin/Hopkins method has also been published for even higher TG levels [9].

One of the basic tenets of lipid-lowering therapy is that the greater the risk, the greater the benefit. Hence, more attention should be paid to treating patients at very high cardiovascular risk. The achievement of LDL-C goals, unfortunately, falls short of its potential worldwide, with the worst rates occurring in the very-high-cardiovascular-risk category, which paradoxically has the most stringent targets to reach [10].

The inclusion of ezetimibe is crucial for reaching these targets. An analysis of a Korean randomized clinical trial and health insurance database demonstrated that a combination of a moderate-intensity statin and ezetimibe had a beneficial effect on the incidence of cardiovascular events when compared to high-intensity statin monotherapy. This combination also led to fewer interruptions of statin therapy and fewer new-onset diabetes cases requiring treatment in that group [11,12].

In our current study, we aimed to investigate the effects of the most potent statin, rosuvastatin, as a monotherapy and in combination with ezetimibe. This research draws from the larger nationwide **T**reatment **T**o **T**arget with Rosuvastatin or **F**ree/Fix Comb**I**nation of Rosuvastatin and Ezetimibe for Vascular Protection in Hun**G**arian **H**ypercholesterolemic Patien**T**s (3T-FIGHT) trial, which studied the results of six months of intense lipid-lowering therapy with an escalation of statin therapy or a combination of statin and ezetimibe in Hungarian patients at very high cardiovascular risk who were previously undertreated for dyslipidemia. The objective of the current study was (1) to investigate the achievement of LDL-C goals; (2) to investigate the achievement of non-HDL-C goals; and (3) to compare LDL-C calculated by three different methods (F-LDL-C, MH-LDL-C and S-LDL-C) and analyze their limitations.

## 2. Materials and Methods

### 2.1. Study Design and Patient Enrolment

**Figure 1:**
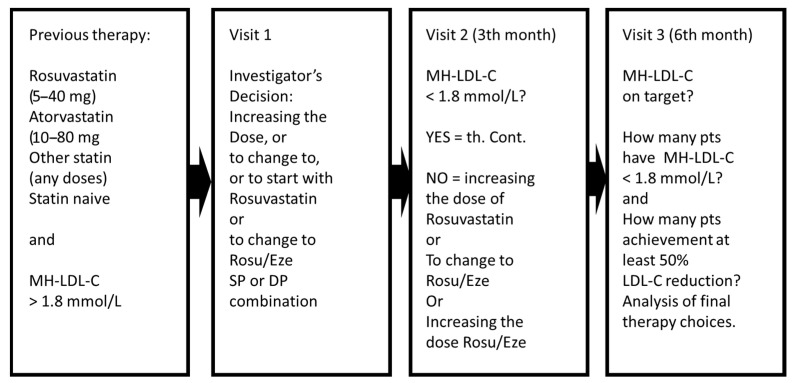
The course of the study.

The 3T-FIGHT trial included patients at very high cardiovascular risk who had LDL-C levels above 1.8 mmol/L before their initial visit with or without lipid-lowering therapy. As exclusion criteria, we used the metabolic and safety parameter limits used in lipid-lowering drug trials in addition to triglyceride levels above 4.5 mmol/L. This prospective observational study was planned between 2018 and 2019 and received approved in 2019, prior to the publication of the latest 2019 lipid guidelines of the European Society of Cardiology and the European Atherosclerosis Society. The study was performed according to the Helsinki Declaration and approved by the Medical Research Council, ETT-TUKEB (registration number: 36772-2/2019; date of approval: 15 July 2019). Participating patients signed a consent form before entering the study. The primary aim was to adjust a more effective lipid-lowering therapy to achieve the target of 1.8 mmol/L LDL-C in patients at very high cardiovascular risk. The study design is illustrated in Figure 1.

The study was conducted on the patients (*n* = 3017) of 150 general and 60 specialist practices during the different waves of the COVID-19 pandemic in 2020–2021 in Hungary. Given that a significant number of participating general practitioners lacked the funding to order direct LDL-C measurements, we uniformly assessed LDL-C levels at each visit based on uniformly calculated values (MH-LDL-C).

In addition to lipid profiles (total cholesterol [TC], HDL-C, TG), we measured CK, AST, ALT, eGFR, hsCRP, blood glucose, and HbA1C as safety parameters. The tests were performed in accredited laboratories within Hungary.

In the present analysis, MH-LDL-C was the primary outcome measure used to assess treatment outcome and the need for further follow-up, but F-LDL-C and S-LDL-C were also calculated at each visit. For F-LDL-C, we used the formula TC–HDL-C–TG/2.2 [6]; for MH-LDL-C, the formula was TC–HDL-C − TG/(TG and non-HDL-C dependent ratios) [7]; and for S-LDL-C, the formula was (TC/0.948) − (HDL-C/0.971) − ((TG/8.56) + (TGxnon-HDL-C/2140) − (TG^2^/16100)) − 9.44 [8,9]. Non-HDL cholesterol (non-HDL-C) was obtained by subtracting HDL-C from total cholesterol. For the target values, we took into account the recommendations of the current European guidelines, adding 0.8 to the LDL-C target value to determine the non-HDL-C target [10].

At the first visit (V1), investigators decided whether to initiate rosuvastatin monotherapy or rosuvastatin/ezetimibe combination therapy for the subsequent three months based on an MH-LDL-C value (exceeding 1.8 mmol/L). At the second visit (V2), if the patient reached the target MH-LDL-C of 1.8 mmol/L, the previous therapy was continued. If not achieved, then at the investigator’s discretion, either the rosuvastatin dose was increased or some rosuvastatin/ezetimibe combination was started for the next three months. After month six of the study, we analyzed at the V3 visit the portion of patients in each treatment arm who achieved the target range below 1.8 mmol/L and/or at least a 50% reduction in MH-LDL-C. As the test results were evaluated in 2022, we also naturally assessed the achievement rates for the more stringent post-2019 target of 1.4 mmol/L.

### 2.2. Statistical Analyses

Statistical analysis was performed using SPSS 23.0 (Armonk, NY, USA). The normality of variables was checked using the Kolmogorov–Smirnov test. We performed a paired *T*-test for normally distributed variables and the Wilcoxon matched pairs test for skewed variables to detect the statistical differences after the 6-month treatment. Data are presented as the mean ± standard deviation or median (lower–upper quartiles). Goal achievement rates for LDL cholesterol goals of 1.8 and 1.4 mmol/L and non-HDL cholesterol goals of 2.6 and 2.2 were calculated by the three methods, F-LDL-C, MH-LDL-C, and S-LDL-C, respectively. The statistical difference between categorical variables was analyzed using the chi-squared test. A *p*-value ≤ 0.05 was considered statistically significant.

## 3. Results

Patient characteristics and main laboratory parameters of the enrolled subjects are presented in Table 1. The mean age of the 3017 patients included in the study was 65 ± 10 years—53% were men (64 ± 10 years) and 47% were women (66 ± 10 years). The mean MH-LDL-C value significantly decreased from 3.64 ± 1.05 mmol/L at baseline to 2.03 ± 0.79 mmol/L at the end of the study (*p* < 0.001), representing an average reduction of 44%. Patient co-morbidities at enrolment were as follows: cardiovascular disease (ACS, AMI, CABG, stent) in 55.5%; cerebrovascular disease (stroke, TIA) in 35.6%; peripheral atherosclerosis (PAD) in 20.4%; hypertension in 86.1%; diabetes in 43.4%; and chronic kidney disease in 2.9%.

At the first visit, eight percent of patients (*n* = 241) were statin-naïve. Of those already on statin therapy, 53% (*n* = 1599) were taking rosuvastatin, 34% (*n* = 1026) were on atorvastatin, and 5% (*n* = 151) were on other statin monotherapies. Specifically, 13% of patients were on 40 mg of rosuvastatin, 4% on 30 mg, 57% on 20 mg, and 1.5%, 3.6%, and 46% of patients were taking 80, 60, and 40 mg of atorvastatin, respectively. In total, 56% of the patients included (*n* = 1727) were on high-intensity statin therapy. By the end of the study, 20.5% of patients were receiving rosuvastatin monotherapy (315 patients (51%) received 40 mg and 197 patients (32%) received 20 mg), 63.7% were on a fixed-dose combination (1479 patients (77%) received a 20/10 mg formulation), and 15.7% were receiving a two-tablet rosuvastatin/ezetimibe combination (257 patients (57%) were taking the 40/10 mg dose) (Table 1).

The three ways of calculating LDL-C values and the achievement rates of LDL-C goals 1.8 and 1.4 mmol/L and non-HDL-C goals are shown in Table 2. In the overall study population, the achievement rate for non-HDL-C targets was significantly higher (*p* < 0.001) compared to F-LDL-C goals. This rate was similar to the achievement rates of LDL-C goals calculated using either the Martin–Hopkins or Sampson formulas. At TG levels below 1.2 mmol/L, the difference was even more pronounced in favor of non-HDL-C goal achievement. When looking at the TG range between 1.2 and 1.69 mmol/L, the MH-LDL-C and S-LDL-C goal achievement was equal, and there was also a higher success rate for F-LDL-C and non-HDL-C goal achievement. At TG levels between 1.7 and 2.29 mmol/L, significantly (*p* < 0.001) more patients were in the target range when LDL-C was calculated using the Friedewald method. This “false” success became more pronounced when TG levels exceeded 2.3 mmol/L.

Of course, higher TG levels were more prevalent in diabetic patients. At the V1 visit, a TG level of 2.3 mmol/L or higher occurred in 42% of diabetic patients, 27.4% of non-diabetic patients, and 34% of all patients. By the final visit, the prevalence was 10% in diabetic patients, 6.4% in non-diabetic patients, and 8% in all patients.

**Figure 2:**
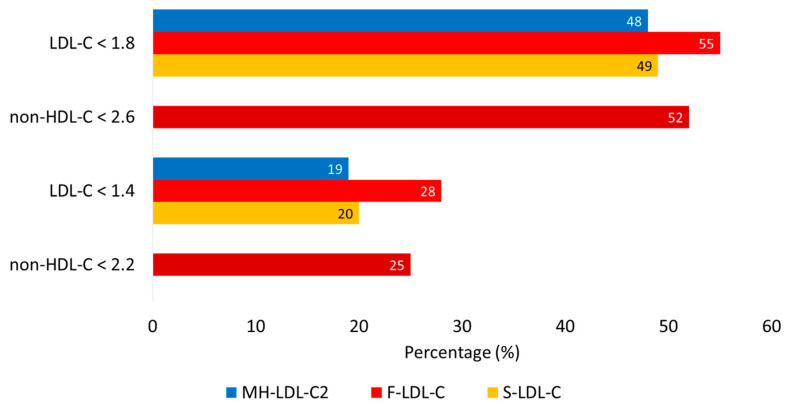
Goal achievement rates for LDL cholesterol goals of 1.8 and 1.4 mmol/L and non-HDL cholesterol goals of 2.6 and 2.2 calculated by the three methods. Abbreviations: F-LDL-C, Friedevald low-density lipoprotein cholesterol; HDL-C, high-density lipoprotein; MH-LDL-C, Martin/Hopkins low-density lipoprotein cholesterol; S-LDL-C, Sampson low-density lipoprotein cholesterol.

Table 3 illustrates the average LDL-C values and goal achievement rates calculated by the three methods for the groups of patients receiving rosuvastatin monotherapy and the rosuvastatin/ezetimibe combination. The table clearly shows that a significantly higher number of patients achieved the recommended LDL-C goals in the groups receiving the rosuvastatin/ezetimibe combination (*p* < 0.05), with the most pronounced effect in those on the fixed combination. The average LDL-C values achieved were also in line with this. Figure 2 further visualizes the achievement rates for LDL-C goals of 1.8 and 1.4 mmol/L calculated by the three methods and non-HDL-C goals of 2.6 and 2.2 mmol/L. Table 4 details the achievement rates of the non-HDL-C targets of 2.6 and 2.2 mmol/L by LDL-C calculated by the three methods in different groups of patients with different triglyceride levels (below 1.2 mmol/L, between 1.2 and 1.69 mmol/L, between 1.7 and 2.29 mmol/L, and above 2.3 mmol/L). The table reveals that in the TG range above 2.3 mmol/L, if non-HDL-C is at the goal (either below 2.6 or 2.2 mmol/L), then all three LDL-C calculations also indicate that LDL-C is at the target (below 1.8 or 1.4 mmol/L). In such cases, the non-HDL-C–LDL-C difference is certainly at least 0.8 mmol/L). In the TG range between 1.7 and 2.29 mmol/L, it is only true for F-LDL-C, with target achievement rates significantly lower for MH-LDL-C and S-LDL-C calculations (*p* < 0.001). At TG levels between 1.2 and 1.69 mmol/L, the difference is less than 0.8 mmol/L in 20–40% of cases. For TG levels below 1.2 mmol/L, all three LDL-C calculations show only about a two-thirds goal achievement rate (the difference between LDL-C goal achievement rates obtained by the three methods was not significant).

## 4. Discussion

Lipid lowering is a cornerstone of cardiovascular prevention, with the achieved LDL-C level serving as a key indicator of its effectiveness. The gold standard method for LDL-C determination would be the precipitation ultracentrifugation method, but due to the prohibitive financial costs, these methods have not been widely used in daily clinical practice. Consequently, the laboratory determination of D-LDL-C via the homogeneous enzyme method has become globally accepted. However, even this is not always feasible, often forcing clinicians to rely on calculated LDL-C values. The F-LDL-C was used the longest and perhaps is still the most commonly used method [6]. Recognizing its inherent errors, MH-LDL-C and S-LDL-C calculation methods were subsequently developed [7,8].

The 3T-FIGHT multicenter Hungarian study enrolled 3017 patients at very high cardiovascular risk. Crucially, the study was designed before the publication of the current European cholesterol recommendations (2019), necessitating the consideration of both older and newer LDL-C targets. As direct LDL-C measurement was not possible at most sites, calculated values were primarily used, with treatment decisions at V1 and V2 visits based on MH-LDL-C. We also calculated F-LDL-C and S-LDL-C for comparative analysis. We found no significant difference between MH-LDL-C and S-LDL-C values, but both were consistently and significantly higher than F-LDL (by an average of over 10%, *p* < 0.05). The same trend was observed in goal achievement rates using these methods. The practical implication for daily practice, particularly in Hungary where F-LDL-C is most commonly used, is significant: a 15–20% lower F-LDL-C might lead clinicians to believe therapy intensification is unnecessary, potentially disadvantaging patients who, in reality, have higher true LDL-C levels.

Our own prior analyses have similarly shown that even with fasting triglyceride levels as low as 1.0 mmol/L (with a rate of deviation increasing in parallel with triglyceride level), F-LDL-C was found to be 0.3–1.0 mmol/L lower than D-LDL. This discrepancy can have serious implications for therapeutic decisions, potentially delaying the initiation of more intensive statin and/or combination lipid-lowering therapy [12].

Globally, LDL-C remains our primary tool for assessing a patient’s cardiovascular risk from a lipid perspective. However, we knowledge that a better marker for this would be the determination of ApoB [13].

Interestingly, a previous meta-analysis of statin trials by Boekholdt et al. demonstrated that non-HDL-C levels were more strongly associated with the future incidence of cardiovascular events than either LDL-C or ApoB in patients receiving statin-based therapy [14].

Furthermore, a state-of-the-art peer review published in 2023 considers non-HDL-C levels an independent risk factor with practical utility in assessing residual risk in patients on statin therapy, especially in the presence of obesity, diabetes, or other metabolic abnormalities [15]. Significant abnormalities in lipid profiles may emerge with TG levels as low as 2.3 mmol/L. This statement also recommends that country-specific targets for non-HDL-C should be established [16], considering local trends and geographical variations in average non-HDL-C levels [17,18]. Currently, Hungary aligns its non-HDL-C targets with European guidelines.

ApoB tests are not widely available yet, and their cost will certainly complicate their broader use. In Hungary, ApoB testing is mostly limited to vascular disease specialists in university laboratories and private networks. In contrast, calculating non-HDL-C does not cost extra. Its wider use may be facilitated by the SCORE2 risk classification in the 2021 European Preventive Guidelines. These and the 2019 ESC/EAS cholesterol guidelines recommend adding 0.8 (in mmol/L) to the LDL-C goal to determine non-HDL-C goals [5,10].

Table 4 shows the non-HDL-C goal achievement rates as a function of triglyceride levels for LDL-C calculated by the three methods. The lower the TG level, the more significant the difference becomes between the calculated LDL-C and the non-HDL-C goals. This discrepancy is largely attributed to remnant cholesterol (RC), which is highly dependent on TG. As shown in Table 4, only at TG levels above 2.3 mmol/L will non-HDL-C consistently be at the goal (below 2.2 or 2.6 mmol/L) when LDL-C is also at the goal (below 1.4 or 1.8 mmol/L).

At TG levels between 1.2 and 1.69 mmol/L, non-HDL-C can no longer be a reliable substitute for LDL-C. In 20–40% of cases, regardless of the calculation method, the difference between LDL-C and non-HDL-C is less than 0.8 mmol/L. Therefore, it can be concluded that at TG levels below 1.7 mmol/L, the difference of 0.8 between LDL-C and non-HDL-C is not true in any case. It is important to note that this applies specifically to calculated LDL-C values, as D-LDL-C was not available in our study.

The use of non-HDL-C was previously recommended primarily for conditions with high TG levels (such as diabetes mellitus, metabolic syndrome, and chronic renal failure), and its general use has recently been revealed. It forms the basis for lipid risk assessment in the SCORE2 system, as outlined in the 2021 European guidelines for cardiovascular prevention [5]. Schubert et al. have used it in monitoring lipid results in post-myocardial infarction patients [19], and a meta-analysis of 11,000 patient results has even suggested its use as a primary endpoint [20]. The general use of non-HDL-C may be argued against by the fact that calculated LDL-C levels appear to be inaccurate in the low TG ranges. Undeniably, our conclusion is not based on D-LDL-C levels, and therefore confirmation of this observation in a large number of patients with D-LDL-C measurements is warranted.

The limitations of our study include its non-blinded design and the investigator-led randomization into two arms. A significant disadvantage is that D-LDL determination was not possible at most sites, which forced us to use calculated values, and our analyses are based on LDL-C calculated in different ways. Nevertheless, a major strength of our work was that it was performed on a nationally representative sample, offering a real-world snapshot of lipid-lowering practices in Hungary during 2020–2021. This is particularly relevant given that in Hungary, general practitioners can only prescribe ezetimibe or ezetimibe-containing lipid-lowering combinations to their patients upon recommendation. The 3T-FIGHT trial clearly demonstrates that using an intensive statin/ezetimibe combination achieves a higher proportion of the recommended LDL-C and non-HDL-C goals provided that patients take the prescribed medication. We plan to share the insights gained from our study with general practitioners and specialist colleagues nationwide, aiming to promote more informed and effective lipid-lowering therapy for enhanced vascular protection for our patients.

## 5. Conclusions

Based on our study, the accuracy of the universal practice of adding 0.8 mmol/L to the LDL-C goal to determine the non-HDL-C goal is questionable. This relationship appears to be dependent on TG levels: while valid for higher TG levels, the accuracy of this 0.8 mmol/L addition decreases as TG levels get lower. When a D-LDL-C measurement is not available, we believe that the use of the MH-LDL-C calculation is the most appropriate approach. This method is available via phone applications, is more widely adopted, and was introduced earlier than the Sampson method, with no significant difference observed between the two. Furthermore, when TG levels are high (typically above 2.3 mmol/L), the practical use of non-HDL-C is also justified for better patient management.

## Figures and Tables

**Table 1:** Patient characteristics and laboratory parameters during the three visits and the lipid-lowering treatments used at baseline and during the study.

	V1	V2	V3	*p*-Values (V1 vs. V3)
**Patient number; *n***	3017			
**Female; *n*(%)**	1405 (47)			
**Male; *n*(%)**	1612 (53)			
**Age (yrs)**	65 ± 10			
**Total cholesterol (mmol/L)**	5.87 ± 1.19	4.44 ± 1.04	4.11 ± 0.87	<0.001
**HDL-C (mmol/L)**	1.41 ± 0.44	1.46 ± 0.42	1.47 ± 0.39	<0.001
**Non-HDL-C (mmol/L)**	4.46 ± 1.15	2.98 ± 1.00	2.64 ± 0.85	<0.001
**Triglyceride (mmol/L)**	2.08 ± 0.89	1.73 ± 0.62	1.61 ± 0.54	<0.001
**MH-LDL-C (mmol/L)**	3.64 ± 1.05	2.33 ± 0.92	2.04 ± 0.78	<0.001
**F-LDL-C (mmol/L)**	3.52 ± 1.12	2.20 ± 0.96	1.91 ± 0.81	<0.001
**S-LDL-C (mmol/L)**	3.61 ± 1.06	2.29 ± 0.94	2.00 ± 0.81	<0.001
**Remnant cholesterol (mmol/L)**	0.91 ± 0.26	0.65 ± 0.17	0.60 ± 0.14	<0.001
**eGFR (mL/min/1.73 m^2^)**	77.0 ± 11.6	77.6 ± 11.2	78.4 ± 18.8	<0.001
**TSH (mIU/L)**	2.27 ± 0.96	-	-	
**Hemoglobin A1c (%)**	6.25 ± 1.02	6.21 ± 0.92	6.27 ± 0.89	<0.001
**hsCRP (mg/L)**	6.43 ± 5.1	5.21 ± 3.4	5.23 ± 3.5	<0.001
**AST (U/L)**	27 ± 13	26 ± 10	26 ± 10	0.002
**ALT (U/L)**	28 ± 15	28 ± 13	27 ± 11	0.009
**Creatine kinase (IU/L)**	89 ± 51	87 ± 50	88 ± 49	0.322
**Rosuvastatin *n* (%); 5–15 mg**	416 (14)	52 (2)	42 (1.5)	
**Rosuvastatin *n* (%); 20–40 mg**	1183 (59)	605 (20)	575 (19)	
**Atorvastatin *n* (%); 10–30 mg**	503 (17)	0	0	
**Atorvastatin *n* (%); 40–80 mg**	523 (17)	0	0	
**Other statin *n* (%)**	151 (5)	0	0	
**Statin naive *n* (%)**	241 (8)	-	-	
**Rosuvastatin/eze (SP)**	-	1940 (64)	1922 (63.7)	
**Rosuvastatin/eze (DP)**	-	420 (14)	477 (15.7)	

Abbreviations: ALT, alanine transaminase; AST, aspartate transferase; DP, double pill-free combination; eGFR, estimated glomerular filtration rate; F-LDL-C, Friedevald low-density lipoprotein cholesterol; HDL-C, high-density lipoprotein; hsCRP, high-sensitivity C-reactive protein; MH-LDL-C, Martin/Hopkins low-density lipoprotein cholesterol; S-LDL-C, Sampson low-density lipoprotein cholesterol; SP, single pill-fixed combination; TSH, thyroid-stimulating hormone.

**Table 2:** Average values of LDL-cholesterol calculated by the three methods and non-HDL-cholesterol and their goal achievement rates based on triglyceride quartiles.

	All	TG < 1.2	TG 1.2–1.69	TG 1.7–2.29	TG ≥ 2.3
**Patients *n* (%)**	3017	523 (17)	1274 (42)	981 (33)	239 (8)
**MH-LDL-C**	2.04 ± 0.78	1.85 ± 0.69	2.04 ± 0.69	2.08 ± 0.79	2.23 ± 1.20
**<1.8 mmol/L**	48%	55%	45%	48%	47%
**<1.4 mmol/L**	19%	28%	15%	18%	24%
**F-LDL-C**	1.91 ± 0.81	1.83 ± 0.70	1.96 ± 0.71	1.89 ± 0.84	1.82 ± 1.29
**<1.8 mmol/L**	55% *	57%	51% *	58% *	60% *
**<1.4 mmol/L**	28% *	29%	20% *	31% *	49% *
**S-LDL-C**	2.00 ± 0.81	1.86 ± 0.72	2.03 ± 0.71	2.02 ± 0.82	2.02 ± 1.26
**<1.8 mmol/L**	49%	53%	45%	51%	55%
**<1.4 mmol/L**	20%	26%	15%	22%	32%
**∆MH-F-LDL-C**	7% 9%	2% 1%	6% 5%	10% 13%	13% 25%
**∆MH-S-LDL-C**	1% 1%	2% 2%	0% 0%	3% 4%	8% 8%
**∆F-S-LDL-C**	6% 8%	4% 3%	6% 5%	7% 9%	5% 17%
**non-HDL-C**	2.54 ± 0.85	2.29 ± 0.71	2.61 ± 0.72	2.75 ± 0.84	3.15 ± 1.37
**<2.6 mmol/L**	57% *	72% *	57% *	53%	42%
**<2.2 mmol/L**	28% *	46% *	26% *	24%	21%

Abbreviations: F-LDL-C, Friedevald low-density lipoprotein cholesterol; HDL-C, high-density lipoprotein; MH-LDL-C, Martin/Hopkins low-density lipoprotein cholesterol; S-LDL-C, Sampson low-density lipoprotein cholesterol; TG, triglyceride. * *p* < 0.001 MH-LDL-C and S-LDL-C goal achievement vs. F-LDL-C and non-HDL-C goal achievement.

**Table 3:** Average values of LDL cholesterol calculated by the three methods and their goal achievement rates in groups of patients receiving rosuvastatin monotherapy and the rosuvastatin/ezetimibe combination.

	All	Rosu	SP Rosu/Eze	DP Rosu/Eze
**Patients *n* (%)**	3017	618 (20)	1922 (64)	477 (16)
**MH-LDL-C**	2.04 ± 0.78	2.16 ± 0.70	1.99 ± 0.79	2.07 ± 0.79
**<1.8 mmol/L**	48%	37%	52% *	45% *
**<1.4 mmol/L**	19%	11%	22% *	18% *
**F-LDL-C**	1.91 ± 0.81	2.05 ± 0.74	1.86 ± 0.83	1.94 ± 0.82
**<1.8 mmol/L**	55%	46%	58% *	54% *
**<1.4 mmol/L**	28%	17%	31% *	28% *
**S-LDL-C**	2.00 ± 0.81	2.13 ± 0.73	1.95 ± 0.82	2.03 ± 0.81
**<1.8 mmol/L**	49%	38%	53% *	47% *
**<1.4 mmol/L**	20%	12%	23% *	19% *
**non-HDL-C**	2.54 ± 0.85	2.76 ± 0.75	2.59 ± 0.87	2.69 ± 0.88
**<2.6 mmol/L**	57%	47%	61% *	56% *
**<2.2 mmol/L**	28%	18%	32% *	26% *

Abbreviations: DP, double pill; F-LDL-C, Friedevald low-density lipoprotein cholesterol; HDL-C, high-density lipoprotein; MH-LDL-C, Martin/Hopkins low-density lipoprotein cholesterol; S-LDL-C, Sampson low-density lipoprotein cholesterol; SP, single pill. * *p* < 0.05 rosuvastatin monotherapy vs. rosuvastatin/ezetimibe.

**Table 4:** Achievement rates of non-HDL cholesterol goals of 2.6 and 2.2 mmol/L as a function of LDL cholesterol goals calculated by the three methods and triglyceride quartiles.

	TG < 1.2	TG 1.2–1.69	TG 1.7–2.29	TG ≥ 2.3
Patients (*n*)	523	1274	981	239
	non-HDL-C < 2.6			
LDL-C < 1.8				
F-LDL-C	78.8% *	89.4% *	100%	100%
MH-LDL-C	76.7% *	78.4% *	89.1% *	100%
S-LDL-C	73.8% *	78.2% *	94.5% *	100%
	non-HDL-C < 2.2			
LDL-C < 1.4				
F-LDL-C	63.2% *	78.2% *	99.6%	100%
MH-LDL-C	61.1% *	60.0% *	76.2% *	100%
S-LDL-C	56.5% *	59.4% *	90.5% *	100%

Abbreviations: F-LDL-C, Friedevald low-density lipoprotein cholesterol; HDL-C, high-density lipoprotein; MH-LDL-C, Martin/Hopkins low-density lipoprotein cholesterol; S-LDL-C, Sampson low-density lipoprotein cholesterol; * *p* < 0.001 non-HDL-C goal achievement vs. LDL-C goal achievement.

## Data Availability

All data generated or analyzed during this study are included in this published article. All data generated or analyzed during the current study are available from the corresponding author upon reasonable request.
